# Intrarater and Interrater Reliability and Concurrent Validity of the 3-Meter Backward Walk Test in Non-frail, Pre-frail, and Frail Older Adults in Residential Care Homes

**DOI:** 10.1093/ptj/pzaf066

**Published:** 2025-05-05

**Authors:** Ali Kapan, Milos Ristic, Andreas Konrad, Thomas Waldhoer

**Affiliations:** Center for Public Health, Department of Social and Preventive Medicine, Medical University of Vienna, 1090 Vienna, Austria; Center for Public Health, Department of Social and Preventive Medicine, Medical University of Vienna, 1090 Vienna, Austria; Institute of Human Movement Science, Sport and Health, Graz University, 8020 Graz, Austria; School of Human Kinetics and Recreation, Memorial University of Newfoundland, St. John’s, NL A1C 5S7, Canada; Center for Public Health, Department of Epidemiology, Medical University of Vienna, 1090 Vienna, Austria

**Keywords:** Accidental Falls, Diagnostic Techniques and Procedures, Geriatrics, Geriatric Assessment

## Abstract

**Importance:**

Assessing backward walking ability in older adults is crucial due to its strong association with balance, mobility, and fall risk. The 3-meter backward walk test (3MBWT) provides a quick and valid tool for this purpose.

**Objective:**

The objective of this study was to assess the reliability and validity of the 3MBWT and to determine its effectiveness in differentiating between different levels of frailty**.**

**Design:**

This study used a cross-section design.

**Setting:**

The study was carried out in residential care homes for older adults.

**Participants:**

Participants were able to walk with or without assistance and were excluded if they had a Mini-Mental State Examination score ≤ 17.

**Interventions:**

Participants underwent the 3MBWT and the 10-meter walk test (10MWT). In addition, the Short Physical Performance Battery (SPPB) was administered.

**Main Outcome(s) and Measure(s):**

Frailty was assessed using the SHARE-FI instrument, with participants classified as non-frail, pre-frail, or frail. Reliability of the 3MBWT was assessed using intraclass correlation coefficient (ICC [3,1]), and concurrent validity was assessed using the 10MWT and SPPB. Fall history over the past year was obtained from medical records and participant interviews.

**Results:**

Participants (*n* = 217) were categorized as non-frail (27%), pre-frail (31%), or frail (42%). The average 3MBWT times were 5.0, 5.9, and 11.8 seconds for participants who were non-frail, pre-frail, and frail, respectively. The 3MBWT showed good to excellent intrarater reliability (ICC = 0.89–0.93) and excellent interrater reliability (ICC = 0.90–0.99). It showed strong correlations with the 10MWT (r = 0.91) and SPPB walking speed (*r* = 0.94), and a negative correlation with the SPPB total score (*r* = −0.86). Moderate correlations were found with the Falls Efficacy Scale International (*r* = 0.71) and Multidimensional Fatigue Inventory (*r* = 0.61), and a high correlation with falls in the last 12 months (*r* = 0.74).

**Conclusions:**

The 3MBWT is a reliable tool for assessing physical performance and differentiating levels of frailty in older adults, with a strong association with fall history.

**Relevance:**

The 3MBWT offers clinicians a quick, simple, and valid tool for assessing older adults in residential care.

## INTRODUCTION

Frailty significantly affects balance and increases the risk of falls, especially in older adults. A systematic review and meta-analysis shows that frail people have a 48% higher risk of falling than non-frail people. The incidence of falls among the frail is approximately twice that of females, representing a 44% increased risk.^1^ This heightened risk is attributed to factors such as muscle weakness, impaired balance, reduced visual and sensory acuity, and overall diminished physical functionality.^2^^,^^3^ Given the high risk of falls, particularly in nursing homes, the use of rapid, valid, and reliable balance assessment tools is critical.^4^^,^^5^ These tools can help health care professionals address the physiological factors that contribute to falls and implement effective prevention strategies. Currently, a number of tests are used to assess balance and mobility in this population. Commonly used rapid assessments include the Timed Up and Go (TUG),^6^ the 10-Meter Walk Test (10MWT),^7^ the Short Physical Performance Battery Test (SPPB),^8^ and the Functional Reach Test.^9^ In addition, the Falls Efficacy Scale International (FES-I),^10^ the Modified Fatigue Impact Scale (MFIS),^11^ and general measures of fall risk (such as a history of falls) were used to assess balance and mobility.^12^ These assessments mainly evaluate forward walking, backward turning, and stepping ability but do not include backward walking performance. While they provide important information about functional mobility and fall risk, meta-analyses of these measures indicate their poor ability to distinguish between fallers and non-fallers, limited sensitivity, and low predictive validity.^6^^,^^13^^,^^14^

Studies show that backward walking is a complex motor task that requires enhanced neuromuscular control and cognitive processing. Backward locomotion is characterized by the absence of peripheral visual feedback and optic flow typically used in planning forward movement.^15^ Due to this lack of visual information, much more proprioceptive feedback is required to control the sequence of steps.^16^ In addition to the visual and proprioceptive demands of postural control, walking backwards also requires increased attention and coordination. The unfamiliar direction of movement engages different muscle groups more intensely and improves motor flexibility.^17^ By challenging balance and stability, backward walking can help improve overall gait safety and reduce the risk of falls.^18^ Therefore, walking backwards should also be considered as a form of training, as a well-trained proprioceptive system allows the body to respond better to changes in environment and posture, improving balance and reducing the risk of falls.^19^ However, clinical implementation requires careful consideration of safety protocols due to the increased risk of falls during backward walking tasks in older adults when performed without appropriate supervision and environmental controls.^20^

Based on these characteristics, the 3-meter backward walking test (3MBWT) was developed and studied to test dynamic balance, gait speed and fall risk in different populations.^21^ In studies with older adults and individuals with stroke, Parkinson disease, and multiple sclerosis, the 3MBWT has been shown to exhibit superior diagnostic accuracy in comparison to the fall assessment methods that have gained prominence in recent years.^22–25^ Furthermore, the 3MBWT has been demonstrated to be a valid and reliable tool for assessing balance and mobility in a range of other clinical populations, including those with cerebral palsy, fibromyalgia, hip and knee arthroplasty, and dementia.^21^ The minimal detectable change (MDC) values indicate that the 3MBWT is sensitive to changes, and normative values have been established for these populations.^21^ The high sensitivity of the test may be attributed to its increased reliance on neuromuscular control, proprioception, and protective reflexes, which collectively make it a sensitive indicator of balance impairment and fall risk.^23^ This underscores the potential of the backward gait test to enhance patient management when integrated into a comprehensive mobility assessment protocol.^26^

Despite the promising potential of the 3MBWT, there is a notable lack of studies examining its validity and reliability specifically in frail individuals living in residential care facilities. Residents often face challenges such as reduced mobility, high prevalence of balance disorders, which require tailored and practical assessment tools.^27^ Assessing the reliability of such tools in specific populations is essential, as reliability may vary depending on the specific characteristics and needs of the target population. The aim of this study is to determine the interrater and intrarater reliability of the 3MBWT in non-frail, pre-frail, and frail older adults using the SHARE-FI score (Frailty Instrument for Primary Care of the Survey of Health, Ageing and Retirement in Europe).^28^ In addition, the 3MBWT will be compared with the 10MWT and the SPPB total score including its subcategories (balance test, gait speed test, chair stand test) to assess its concurrent validity. This study aims to fill a significant gap in the literature by providing robust data on the effectiveness of the 3MBWT in assessing physical performance and fall risk in frail older adults, which is essential for the development of targeted interventions and effective fall prevention strategies in clinical settings.

## METHODS

### Study Design and Participants

The study was conducted over a 6-month period, from January to June 2024, in 2 residential care facilities in Vienna.^29^ These Austrian residential care facilities are comparable to a combination of senior housing and assisted living facilities in the United States, as they provide a continuum of care ranging from independent living to nursing care for residents with higher needs. The multi-level care concept integrates independent living with access to comprehensive on-site care services as required. The residential infrastructure includes accessible single and double rooms for couples. Services encompass physical therapy, occupational therapy, and socio-cultural activities. In addition, the facilities offer specialist care for those with special needs such as dementia or visual impairment.

A cross-sectional design was employed for assessing intrarater and interrater reliability and concurrent validity of the 3MBWT. The study adhered to the Declaration of Helsinki and received approval from the Clinical Research Ethics Board of Vienna (EK-23-082-0523).

#### Participants

This study used a convenience sample of older adults living in 2 residential care homes in Vienna, which housed 281 residents during the study period. Of these, 234 (83.3%) met the inclusion criteria, which required participants to be able to walk with or without assistance, to provide signed informed consent, and to understand the questionnaires and physical performance tests in either German or English. The remaining 47 residents (16.7%) were excluded due to cognitive impairment (Mini-Mental State Examination [MMSE] ≤17, *n* = 16), mobility limitations that prevented or generally precluded safe walking (*n* = 19), or insufficient knowledge of German or English (*n* = 12). Of the 234 eligible residents, 217 agreed to participate, representing 77.2% of the total number of residents. The main reason for non-participation among eligible residents was unwillingness to participate in the physical examinations (*n* = 17).

### Sample Size Determination

No formal power analysis was performed for this study. The sample size (*n* = 217) reflects the total number of residents who met the inclusion criteria and agreed to participate. This approach was taken to ensure a comprehensive representation of the eligible population in residential care homes.

### Frailty Status

The frailty status of individuals was assessed using SHARE-FI, a sex-specific tool based on the Fried frailty phenotype criteria.^28^ These criteria assess frailty by unintentional weight loss, fatigue, weakness, slow walking speed and low physical activity, focusing on physical aspects. We used the SPPB instead of walking speed for a more comprehensive assessment of physical function, which improved the accuracy of frailty identification.^30^ Based on these criteria, participants were classified as non-frail if they met none of the criteria, pre-frail if they met 1 or 2, and frail if they met 3 or more.

- Unintentional Weight Loss. Participants were assessed for unintentional weight loss using medical records and interviews, specifically asking if they had lost more than 4.5 kg (10 pounds) or 5% of their body weight in the previous 12 months.
- Fatigue. Fatigue was measured using the Multidimensional Fatigue Inventory (MFI-20), which evaluates general, physical, and mental fatigue. A total score of 57.97 or higher on the MFI-20 indicated significant fatigue and potential frailty for individuals aged 75 and older.^31^
- Weakness. Handgrip strength (HGS) was measured using a digital hand dynamometer (CAMRY, model: SCACAM-EH101), in accordance with standardized procedures.^32^ Participants, seated with elbows flexed at 90 degrees and forearms in neutral position, squeezed the dynamometer as hard as possible for 3 to 5 seconds, with 3 trials per hand. The highest value (kg) from each trial was recorded. Weakness was defined as HGS <27 kg for males and < 16 kg for females according to EWGSOP2 guidelines.^33^
- Physical Performance. The SPPB assessed physical performance through balance tests, a 4-meter walking speed test and a chair stand test. Each component was scored from 0 to 4, with a total score of 0 to 12. A score below 7 indicates an increased risk of disability and mortality and defines individuals as frail, with a sensitivity of 65% to 85% and specificity of 70% to 85%.^30^^,^^34^
- Low Physical Activity. Physical activity was measured using the Physical Activity Scale for the Elderly (PASE), which assesses activities such as walking, exercise, housework, gardening and caregiving. The PASE score, which ranges from 0 to 793, reflects the frequency, duration and intensity of activities over the past week, with higher scores indicating greater physical activity.^35^ Low physical activity thresholds based on Auyeung et al^36^ were 0 to 56.4 for males and 0 to 58.8 for females, indicating frailty.

### Procedure

All measurements were carried out by 2 physical therapists, each with more than 5 years' experience in geriatric assessment and rehabilitation. During the measurements, the 2 raters (rater 1 and rater 2) were randomly assigned to ensure that their assignment to participants and measurements was random and unbiased. Thus, if rater 1 was randomly selected, that rater performed the subsequent tests. Measurements were taken in the morning under identical conditions, including uniform flooring, lighting and room size. Participants were given detailed instructions and 2 practice trials of the 3MBWT to familiarize them with the task, as backward walking is less common and can be challenging, particularly for those using assistive devices.

Interrater reliability was calculated from the first trial of the 3MBWT, with measurements recorded simultaneously by both raters using identical stopwatches, without consultation or disclosure of times. After a 90-minute rest period, the randomly selected physical therapist repeated the 3MBWT to determine intrarater reliability. Additional tests included the 10MWT, the SPPB, and the HGS test. During the 60 to 90 minute break, participants completed questionnaires: FES-I, PASE, MFI.

### Measurements

#### 3-Meter Backward Test, 4-Meter Walk Test (SPPB), and 10-Meter Walk Test

**Figure 1:**
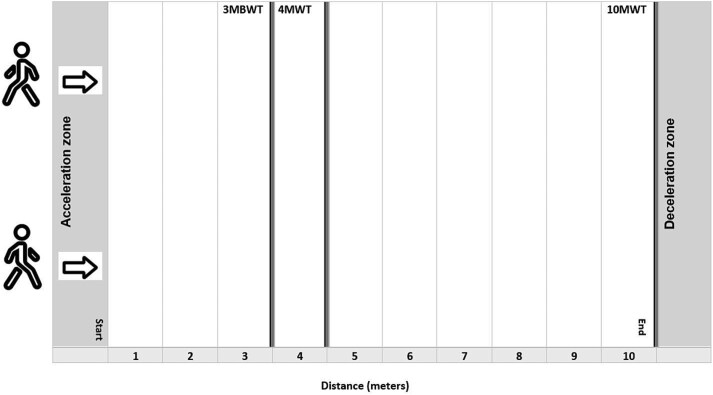
The Image Illustrates the Setup and Protocol for the 3MBWT, SPPB, and 10MWT Tests. The standardized 12-meter test track includes a 1-meter acceleration zone at the start and a 1-meter deceleration zone at the end. The individual test sections (3, 4, and 10 m) are clearly marked with black tape to ensure consistency in measurements. Abbreviations: 3MBWT = 3-Meter Backward Walk Test; 4MWT = 4-Meter Walk Test (gait speed component of the short physical performance battery); 10MWT = 10-Meter Walk Test.

For the 3 walking tests (3MBWT, SPPB, and 10MWT), a standardized 12-m test course was prepared. This course included a 1-m acceleration zone at the start and a deceleration zone after 11 m (Figure 1). The specific test distances (3, 4, and 10 m) were marked with black tape on the ground. All 3 measurements were conducted according to a strictly standardized protocol, and the commands and instructions given to the participants were identical for all tests. Participants were instructed to “walk as fast as you can, but safely.” Running was prohibited during the trials. For the starting position, they stood at the beginning of the acceleration zone and positioned either their heels (for the 3MBWT) or their toes (for the SPPB and 10MWT) on the starting line. Timing began uniformly after the standardized start command (“1, 2, 3, go”) when the first foot completely crossed the start line after the acceleration zone and stopped when the first foot completely crossed the finish line. Participants were allowed to use their usual walking aids if necessary. To ensure consistency, the same walking aid had to be used for all 3 tests and for the retest. This uniform procedure, including identical instructions and commands, ensured consistency of results and comparability between tests.

#### The Falls Efficacy Scale-International and Falls

The FES-I assesses fear of falling in older adults by evaluating concern during daily activities. It consists of 16 items, scored from 1 (“not at all concerned”) to 4 (``very concerned''), with total scores ranging from 16 to 64. Higher scores indicate more fear of falling.^10^ Falls in the previous 12 months were recorded using the Cogvis fall detection system and recorded in residents' medical records. This non-invasive 3D sensor system is installed in residents' rooms and common areas and detects falls in real time without the need for wearable devices. In addition, participants were also asked whether they had fallen in the previous 12 months and how often. A fall was defined as an event where a person inadvertently comes to rest on the ground, floor, or a lower level. This definition excludes falls caused by overwhelming external forces or medical events such as strokes or seizures.^37^

### Other Variables

The following characteristics were obtained from medical records: age, sex, body mass index (BMI), comorbidities (Charlson Comorbidity Index), and current medications. Additionally, the use of walking aids (ie, walking without an aid or with a cane, quadripod, rollator, or walking frames) was documented based on the aid used in the 3MBWT.

### Statistics

Statistical software SPSS (version 27; SPSS Inc.) was used for analysis. Descriptive statistics summarized sample characteristics. Normality of data was assessed using the Shapiro–Wilk test, histograms and box plots. Differences between non-frail, pre-frail, and frail groups were analyzed using 1-way analysis of variance (ANOVA for normally distributed data with equal variances and the Kruskal-Wallis test for non-normally distributed or unequal variance data). Levene test was used to assess homogeneity of variance for the ANOVA. When ANOVA yielded significant results (*P* < .05), we conducted post-hoc analyses using Tukey test for homogeneous variances and Games-Howell for inhomogeneous variances. Categorical variables were compared using the χ-squared test.

Intrarater and interrater reliability for the 3MBWT was assessed using the intraclass correlation coefficient (ICC) with a 1-way random effects model (ICC [3,1]) to assess the reliability of the measurements. An ICC ≥ 0.9 was defined as excellent, 0.75 to 0.89 as good, 0.5 to 0.74 as moderate, and < 0.5 as poor validity.^38^ Additional calculations included the standard error of measurement (SEM = SD × $\sqrt{\left[1-\mathrm{ICC}\right]}$) and the minimal detectable change (MDC) at the 95% confidence level (MDC95 = SEM × 1.96 × $\sqrt{2}$). Here, 1.96 corresponds to the 95% confidence interval, while $\sqrt{2}$ accounts for the random error introduced by repeated measurements.^39^ Bland-Altman plots were generated to visualize bias and outliers and to show the agreement between measurements.^40^ The concurrent validity was evaluated through the correlation between the 3MBWT and the total SPPB score and with its subcategories (SPPB Balance, SPPB Gait Speed, and SPPB Chair Stand Test), as well as the 10MWT, using either Pearson (r) or Spearman (ρ) correlation coefficients, as appropriate. To further explore the relationship between the 3MBWT, general physical function and health status, correlation coefficients were calculated between the 3MBWT and HGS, falls in the last 12 months, FES-I, PASE and MFI. The coefficients were classified as negligible (0.0–0.30), low (0.31–0.50), moderate (0.51–0.70), high (0.71–0.90) or very high (0.91–1.0).^41^

## RESULTS

The study included 217 participants, of whom 27% were classified as non-frail, 31% as pre-frail, and 42% as frail based on the SHARE-FI assessment. The frail group had a significantly higher mean age compared to the non-frail (9.1 years) and pre-frail (4.4 years) groups. The 3MBWT times differed significantly between groups (non-frail: 5.0 sec, pre-frail: 5.9 sec, frail: 11.8 sec) and aligned with fall history patterns (non-frail: 0%, pre-frail: 24%, frail: 76%), demonstrating the test's ability to discriminate between these groups. ANOVA revealed significant differences, with post-hoc analyses confirming pairwise differences (non-frail vs. pre-frail, non-frail vs. frail, pre-frail vs. frail) for all variables except BMI. Detailed demographic information, disease characteristics and all test results are shown in Table 1.

**Table 1:** Demographic Characteristics and Known Group Validity of the 3 Meter Backward Walk Test and Retrospective Falls*^a^*

Variables	Total (n = 217)	Non-frail (n = 58)	Pre-frail (n = 68)	Frail (n = 91)	*P*
Age, y, mean (SD)	80.0 (4.3)	74.7 (2.1)	79.4 (2.8)	83.8 (3.6)	<.001*^b^*
Sex, n (%)					
Female	143 (65.9)	31 (21.7)	45 (31.5)	67 (46.9)	.024*^c^*
Male	74 (34.1)	27 (36.5)	23 (31.1)	24 (32.4)	
BMI, mean (SD)	24.3 (2.9)	25.1 (1.9)	24.5 (3.1)	23.7 (3.2)	<.001*^b^*
Number of medication, median (IQR)	7.1 (2.8)	5.1 (1.6)	6.2 (2.4)	9.0 (2.4)	<.001*^d^*
Charlson comorbidity, mean (SD)	2.3 (1.2)	1.2 (0.4)	2.0 (0.6)	3.3 (1.1)	<.001*^b^*
Unintentional weight loss, n (%)					
Yes	34 (15.7)	0	8 (8.8)	26 (28.6)	<.001*^c^*
No	183 (84.3)	58 (100.0)	60 (91.2)	65 (71.4)	
FES-I score, mean (SD)	30.0 (14.5)	15.8 (3.3)	23.9 (8.0)	43.6 (10.1)	<.001*^b^*
MMSE score, mean (SD)	27.0 (2.2)	29.2 (0.9)	27.4 (1.3)	25.4 (1.9)	<.001*^b^*
PASE score, mean (SD)	76.3 (52.5)	135.9 (54.2)	82.1 (23.7)	34.0 (15.1)	<.001*^b^*
MFI score, mean (SD)	53.2 (10.6)	40.1 (3.5)	54.3 (8.2)	60.8 (6.5)	<.001*^b^*
SPPB score, mean (SD)	7.9 (3.2)	11.4 (0.8)	9.5 (0.9)	4.6 (1.5)	<.001*^b^*
Balance Test, mean (SD)	2.9 (1.1)	3.8 (0.5)	3.5 (0.6)	1.9 (0.7)	<.001*^b^*
Gait Speed Test, mean (SD)	7.4 (3.7)	4.6 (0.6)	5.7 (1.1)	10.4 (3.8)	<.001*^b^*
Chair Stand Test, mean (SD)	14.2 (5.2)	11.0 (1.1)	12.8 (1.3)	17.2 (6.7)	<.001*^b^*
10MWT, mean (SD)	12.7 (6.0)	8.9 (1.2)	10.5 (1.6)	16.8 (7.3)	<.001*^b^*
Handgrip strength (max), mean (SD)	22.1 (7.3)	28.5 (6.2)	23.3 (5.4)	17.2 (5.5)	<.001*^b^*
Use of walking aids, n (%)					
Unaided	141 (65.0)	58 (100.0)	60 (88.2)	23 (25.3)	<.001*^c^*
Cane	33 (15.2)	0	6 (8.8)	27 (29.7)	
Quadripod	9 (4.1)	0	1 (1.5)	8 (8.8)	
Rollator	32 (14.7)	0	1 (1.5)	31 (34.1)	
Walking frames	2 (0.9)	0	0	2 (2.2)	
Falls in the last 12 mo, n (%)					
Yes	86 (39.6)	0	16 (23.5)	70 (76.9)	<.001*^c^*
No	131 (60.4)	58 (100.0)	52 (76.5)	21 (23.1)	
3MBWT Rater 1 (round 1), mean (SD)	8.1 (4.3)	5.0 (0.8)	5.9 (1.5)	11.8 (4.3)	<.001*^b^*
3MBWT Rater 1 (round 2), mean (SD)	8.9 (5.2)	5.1 (0.7)	6.2 (1.7)	13.3 (5.3)	<.001*^b^*
3MBWT Rater 2, mean (SD)	8.2 (4.3)	5.0 (0.7)	6.1 (1.5)	11.9 (4.2)	<.001*^b^*

^a^ 3MBWT = 3-Meter Backward Walk Test; 10MWT = 10-Meter Walk Test; BMI = body mass index; FES-I = Falls Efficacy Scale International; IQR = interquartile range; MFI = Multidimensional Fatigue Inventory; MMSE = Mini-Mental State Examination; PASE = Physical Activity Scale for the Elderly; SPPB = Short Physical Performance Battery.

^b^ One-way analysis of variance in metric data.

^c^ χ^2^ test in categorical data.

^d^ Kruskal-Wallis test used for non-parametric metric data.

As shown in Table 2, the intrarater reliability for the 3MBWT was good to excellent across groups, with non-frail older adults showing an ICC of 0.89, pre-frail individuals an ICC of 0.93, and frail individuals an ICC of 0.93. The corresponding SEM values were 0.23, 0.41 and 1.32 seconds, with MDC95 values of 0.63, 1.14 and 3.67 seconds, respectively. The interrater reliability was excellent, with ICC values ranging from 0.90 to 0.99 across all groups. Detailed SEM and MDC values for both reliability analyses are shown in Table 2.

**Table 2:** Intrarater and Interrater Reliability for the 3MBWT in Non-frail, Pre-frail, and Frail Individuals*^a^*

Tests for Reliability	Test Rounds and Reliability Metrics	Non-frail (n = 58) S	Pre-frail (n = 68) S	Frail (n = 91) S
Intrarater Reliability	Round 1; mean (SD)	4.98 (0.75)	5.93 (1.50)	11.81 (4.25)
	Round 2; mean (SD)	5.09 (0.68)	6.19 (1.66)	13.25 (5.28)
	ICC (95% CI)	0.89 (0.82–0.94)	0.93 (0.86–0.97)	0.93 (0.56–0.98)
	SEM	0.23	0.41	1.32
	MDC_95%_	0.63	1.14	3.67
Interrater Reliability	Rater 1, mean (SD)	4.98 (0.75)	5.93 (1.50)	11.81 (4.25)
	Rater 2, mean (SD)	5.01 (0.67)	6.08 (1.53)	11.91 (4.24)
	ICC (95% CI)	0.90 (0.83–0.94)	0.95 (0.92–0.97)	0.99 (0.98–0.99)
	SEM	0.22	0.33	0.60
	MDC_95%_	0.62	0.91	1.66

^a^ 3MBWT = 3-meter backward walk test; ICC = intraclass correlation coefficient; MDC_95%_ = minimal detectable change at 95% confidence level; SEM = standard error of measurement; S = seconds.

**Figure 2:**
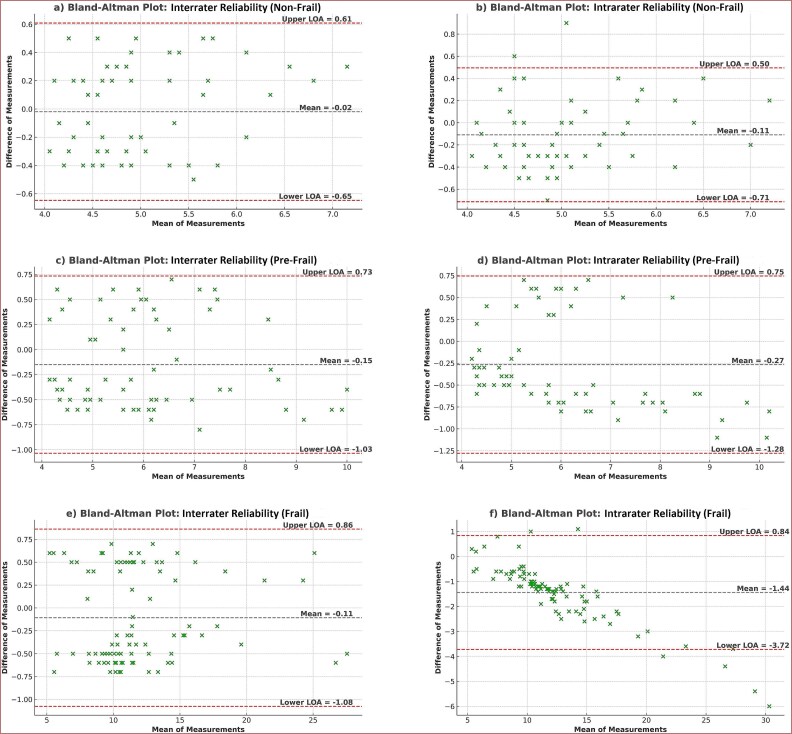
Bland-Altman Plots for Intrarater and Interrater Reliability of the 3-Meter Backward Walk Test in Non-frail, Pre-frail, and Frail Older Adults. The figure shows bland-altman plots for examining interrater and intrarater reliability for different frailty status groups. The central dashed line represents the mean difference, and the outer dashed lines indicate the limits of agreement (LOA). Panels (a)–(e) show the interrater reliability, and panels (b)–(f) show the intrarater reliability for the non-frail, pre-frail, and frail groups, respectively.

Bland-Altman analysis (Figure 2) showed the reproducibility of the 3MBWT across different levels of frailty. For the non-frail group (Figure 2a and b), intrarater reliability had limits of agreement (LOA) from 0.50 to −0.71 with a mean difference of −0.11, while interrater reliability had limits from 0.61 to −0.65 with a mean difference of −0.02. In the pre-frail group (Figure 2c and d), intrarater reliability had limits from 0.75 to −1.28 (mean difference: −0.27), and interrater reliability had limits from 0.73 to −1.03 (mean difference: −0.15). These results indicate minimal systematic bias and good agreement in both groups. The frail group (Figure 2e and f) showed reduced reproducibility, particularly for interrater measurements. Intrarater reliability analysis showed LOA of 0.87 and − 1.08 seconds (mean difference: −0.11), whereas interrater reliability showed significantly wider limits of 0.85 and **−** 3.73 seconds (mean difference: −1.44). This increased variability was particularly evident in participants with 3MBWT times greater than 15 seconds. A detailed visualization of all measures and their distributions can be found in Figure 2.

**Figure 3:**
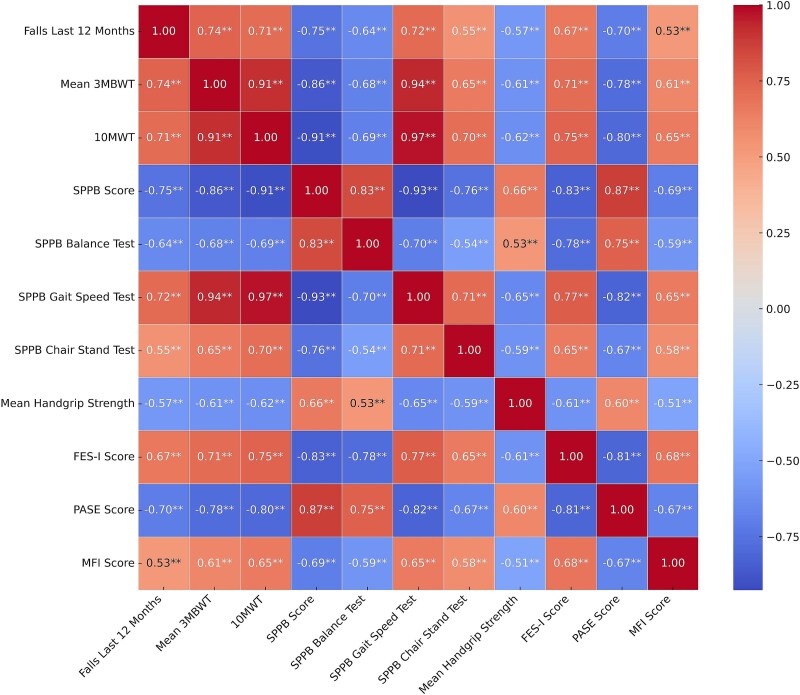
Correlation Heat Map. A heat map with pearson correlation was used for parametric data and spearman correlation for non-parametric data. Abbreviations: 3MBWT = 3-Meter Backward Walk Test; 10MWT = 10-Meter Walk Test; FES-I = Falls Efficacy Scale International; MFI = Multidimensional Fatigue Inventory; PASE = Physical Activity Scale for the Elderly; SPPB = Short Physical Performance Battery; ^**^P ≤.001.

The concurrent validity analysis (Figure 3) shows significantly strongest correlations between the 3MBWT and established gait tests: 10MWT (*r* = 0.91) and SPPB walking speed test (*r* = 0.94). The 3MBWT also showed a strong negative correlation with the SPPB total score (*r* = −0.86) and moderate correlations with its subtests (balance: *r* = −0.68; chair-stand: *r* = 0.65).

**Figure 4:**
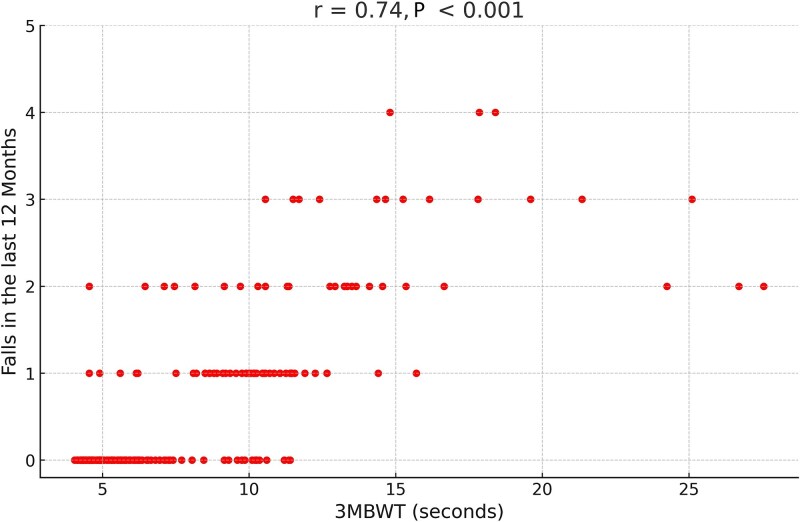
Correlation Analysis With 3MBWT and Falls in the Last 12 Months. The scatterplot shows the relationship (spearman correlation) between the 3-meter backward walk test (3MBWT) times (in seconds) and the number of falls in the last 12 months.

There were also moderate correlations with the FES-I (*r* = 0.71) and MFI (*r* = 0.61) scores. Regarding fall history (Figure 4), the 3MBWT showed a high correlation with falls in the past 12 months (*r* = 0.74), comparable to the 10MWT (*r* = 0.71) and the SPPB score (*r* = −0.75).

## DISCUSSION

This study is the first to examine the reliability and validity of the 3MBWT in individuals with pre-frailty and frailty. The findings indicate that the 3MBWT is a reliable and valid instrument for assessing dynamic balance and gait performance in these populations. In addition, the 3MBWT showed discriminant validity by effectively discriminating between frailty groups and individuals with and without a history of falls. These results highlight the ability of the test to capture clinically meaningful differences in functional status.

Detailed analysis of the measurement properties revealed different levels of reliability between frailty groups. While non-frail and pre-frail groups showed good to acceptable reliability with lower SEM and MDC values, frail individuals showed increased measurement variability. Despite a 90-minute rest period, frail individuals showed a 3MBWT time increase between rounds that was significantly greater than that observed in non-frail individuals. Further examination through Bland-Altman analysis revealed distinct measurement patterns between frailty groups. Non-frail participants showed narrow limits of agreement, indicating consistent test performance. Pre-frail individuals maintained acceptable levels of agreement, albeit with slightly wider limits. The frail group showed significantly increased variability, with particularly wide limits of agreement. This increased variability was primarily due to slower performance times in the second round of testing, particularly for measurements over 15 seconds. The systematic shift towards longer performance times in the second round could be attributed to several factors associated with frailty: physical fatigue, reduced muscle strength and endurance, reduced neuromuscular control, impaired balance, and increased cognitive demands of backward walking.^42^ The complex interaction of these physical, cognitive and functional limitations in frail people appears to affect not only their initial performance but also their ability to maintain consistent performance over repeated measurements. These patterns suggest that measurement stability in frail people is influenced by the multifaceted nature of frailty, and highlight the importance of considering the full clinical picture when interpreting test results in this population. While these variations were observed in repeated measurements, interrater reliability analysis showed strong consistency across all frailty groups, with particularly robust agreement in the frail group. This suggests that the 3MBWT can be reliably administered by different raters regardless of frailty status.

However, several practical considerations affect test performance and interpretation. Individuals who rely on assistive devices for walking may complete the test more slowly than those without assistive devices due to their reliance on the device and the need to use it correctly.^43^ In addition, studies have shown that the weight of assistive devices and the additional cognitive demands of manipulating them while walking backwards can increase physical effort and fatigue, particularly in frail people with reduced upper body strength.^44^ In contrast, people who rely only sporadically on assistive devices, such as very light walking aids, may be able to complete the 3MBWT more quickly when using these devices because they provide greater stability and safety.^45^ This potential improved performance with assistive devices may not fully reflect usual mobility and fall risk without the use of assistive devices, which may confound the results of the assessment. Furthermore, our test protocol included some methodological modifications that warrant discussion. We deviated from the original SPPB in 2 ways: we introduced a 1 m acceleration zone and instructed participants to walk “as fast as safely possible” rather than at their usual pace. While the acceleration zone may provide a more stable measure of walking speed, it may mask clinically relevant information, particularly in older adults with neurological conditions where the initial phase may be diagnostically significant. The instruction to walk faster was chosen to standardize methodology across walking tests, but may limit comparability with studies using the original SPPB protocol. These methodological choices reflect the challenge of balancing standardization with clinical relevance.

When compared with other studies of older adults and patients with stroke, our measures of reliability show a good level of agreement. Previous research has demonstrated the 3MBWT to be reliable in patients with stroke (ICC = 0.97).^26^ In addition, studies reported ICCs of 0.94 to 0.96 for intrarater and 0.96 to 0.99 for interrater reliability in patients with stroke^46^ an intrarater ICC of 0.94 for community-dwelling older adults,^47^ ICCs of 0.96 and 0.97 for older adults with dementia,^48^ and an intrarater ICC of 0.942 for older adults with primary total knee arthroplasty.^49^ Furthermore, a systematic review has corroborated the efficacy and dependability of the backward walking test in other clinical populations, including those afflicted with cerebral palsy, multiple sclerosis, Parkinson disease, and fibromyalgia.^21^ The review emphasized the test's high intrarater, interrater, and reliability, as well as its moderate to substantial concurrent validity.

In addition, our study aimed to assess the concurrent validity of the 3MBWT in relation to various measures of walking speed, mobility and fall risk. As expected, the results showed a strong positive correlation between the 3MBWT and the SPPB speed test (*r* = 0.94) and the 10MWT (*r* = 0.91), indicating that better backward walking performance is associated with better forward walking ability. The strong negative correlation between the total SPPB score and the 3MBWT (*r* = −0.86) was also expected, as lower completion times on the 3MBWT reflect better physical performance, similar to higher SPPB scores. The magnitude of these correlations was stronger than originally hypothesized, suggesting that backward walking may be more closely related to overall functional mobility than previously thought. These findings are consistent with, but show even stronger associations than, previous studies reporting moderate to strong correlations between backward walking tests and assessments of gait speed, balance and mobility, such as the 10MWT, TUG test and Berg Balance Scale.^26^^,^^50^ Our study adds to this evidence base by demonstrating similarly high correlations in a specific population of pre-frail and frail people.

In terms of fall risk, the study found a strong correlation between the 3MBWT and falls in the past 12 months (*r* = 0.74). This correlation is similar to that of the 10MWT (*r* = 0.71, *P* < .001) and the negative correlation with the total SPPB score (*r* = −0.75). These results supported the hypothesis that better performance on the 3MBWT was associated with lower number of falls within the previous 12 months. This highlights the concurrent validity of the 3MBWT with other fall assessments and suggests that it could complement existing tools in providing a more comprehensive assessment of mobility and fall risk in residential care settings. However, further prospective studies are needed to assess its predictive validity for future fall risk.

Although the results are encouraging, it is important to acknowledge the limitations of the study. Notably, the study was conducted in only 2 older adults residential care homes in Vienna, which may limit the generalizability of the results to other settings or populations. Participants with MMSE score ≤ 17 were excluded to ensure participants could follow the study instructions. Hence, our findings may not apply to individuals with significant cognitive impairments. As cognitive decline can occur in older adults and is associated with an increased risk of falls, future studies should include this subgroup for a more comprehensive understanding of the utility of the 3MBWT. A prospective longitudinal study with ongoing fall event monitoring is needed to fully investigate whether the 3MBWT can predict falls in pre-frail and frail older adults.

## CONCLUSIONS

This study showed that the 3MBWT is a reliable and valid tool for assessing balance and gait performance in non-frail, pre-frail, and frail individuals living in residential care homes. The test showed good to excellent intrarater and interrater reliability and strong correlations with established measures of mobility and fall history. These results suggest that the 3MBWT can be used as an alternative brief assessment tool for physical performance. Given its ease of administration and demonstrated concurrent validity, the 3MBWT has potential for integration into routine clinical assessment. Prospective studies are needed to assess its predictive validity for future falls.

## Data Availability

Data are not publicly available due to participant privacy concerns. Anonymized data may be shared upon reasonable request to the corresponding author and pending ethical approval.
